# Developing and integrating a destination decision support algorithm into an innovative electronic communication platform to improve injury care service coordination in Rwanda: the Rwanda912 study protocol

**DOI:** 10.1136/bmjopen-2025-102355

**Published:** 2025-06-27

**Authors:** Rwanda912 RIGHT Group, Justine Davies

**Affiliations:** 1 School of Applied Health Sciences, College of Medicine and Health, University of Birmingham, Birmingham, UK

**Keywords:** ACCIDENT & EMERGENCY MEDICINE, Health informatics, Information technology, Clinical Decision-Making, eHealth

## Abstract

**Introduction:**

Delays in getting injured patients to the hospital in a timely manner can increase avoidable death and disability. Like many low-income or middle-income countries, Rwanda experiences delays related to a lack of efficient prehospital communication and formal guidelines to triage patients for hospital care. This study describes the protocol to develop, roll-out and evaluate the effectiveness of a destination decision support algorithm (DDSA) integrated in an electronic communication platform, ‘912Rwanda’. The DDSA will facilitate the linkage of patients to health facilities able to treat their condition(s).

**Methods and analysis:**

Work will be conducted in the prehospital emergency service ‘Service d'Aide Médicale Urgente’ and health facilities in Kigali city and Musanze district, which serve predominantly urban and rural populations, respectively. We will develop interfaces to capture facility and patient-relevant data, which feed into a guideline-based electronic DDSA to match patients to hospitals. We will assess existing trauma care processes using qualitative and quantitative methodologies. This will be followed by a series of consensus workshops to develop at-scene triage guidelines and agree on variables to capture in the interfaces. The DDSA will be developed based on outputs from these workshops and will be tested against historical ambulance data and expert opinion until acceptable thresholds of performance are achieved. User interfaces will be developed and tested using human–computer interface design principles.

**Discussion:**

The combined collaborative approach of bringing together experts and software developers, and with deep engagement of Rwandan stakeholders, including leadership of Rwanda Ministry of Health through its technical arm, Rwanda Biomedical Center, should lead to an ambulance communication system which is used, sustained and effective.

**Ethics and dissemination:**

The project was approved by the Rwanda National Research Ethics Committee. Annual reports will be disseminated to relevant stakeholders, followed by the public. Publications will be open access as per the funding policy.

**Trial registration number:**

ISRCTN97674565. Registered on 29 July 2024. https://doi.org/10.1186/ISRCTN97674565.

**STRENGTHS AND LIMITATIONS OF THIS STUDY**

- This project holds great promise for improving the efficiency of emergency medical services and ultimately saving lives through the development and integration of a unique, locally developed destination decision support algorithm (DDSA) into a bespoke emergency medical services communication system.
- The documentation of the development process using rigorous methodologies will produce findings that can enhance ambulance software development processes in other similar low-income or middle-income countries.
- The project will feature collaboration across multiple sectors, bringing together academia and industry to deeply understand practical challenges and facilitate the integration of theoretical and practical knowledge.
- We may encounter difficulties due to a lack of high-quality, comprehensive data to inform DDSA development decisions. To address this, we plan to rely on expert practitioner opinions to compensate for any data that is insufficient, incomplete or unavailable.
- The fast-paced changes in software development and technologies may render research findings to become outdated quickly. It will be crucial to keep up with the latest advancements.

## Introduction

 Injuries are a substantial cause of death and disability.^1^ They have a profoundly negative effect on both individuals and society.^2^ Injuries cause about 4.4 million deaths globally, with tens of millions more suffering from non-fatal injuries each year.^1^ Adults of working age in low-income and middle-income countries (LMICs) are primarily and disproportionately affected by injuries and trauma, resulting in severe physical impairment, long-term disability and psychological suffering.^3^

A pillar of high-quality emergency care involves getting patients to the right health facility at the right time.^4^ For severely injured patients, this should ideally be within 1–2 hours; a longer prehospital time is associated with increased mortality.^5^,^7^ This is exemplified in patients with fractures in 18 LMICs, where 72% experienced delays for more than 2 hours before reaching the hospital.^8^ We have found that 40% of deaths after injury are avoidable, and 40% of these were due to delays in getting to facilities.^9^,^11^ The same holds for patients with time-critical conditions.^12^

In Rwanda, injury causes 9% of all deaths; 47% of these occur prehospital and 49% are within the first 24 hours of admission.^13^,^15^ Road traffic incidents (RTIs) are a particular issue; in 2019, the Rwanda National Police documented 4661 injuries and 700 fatalities owing to RTIs.^16^ Of these, 35.6% had lifetime injuries, and around 50% had orthopaedic issues.^16^ As in many other LMICs, there are substantial delays in reaching treatment facilities in Rwanda.^17^,^19^

In 2007, the Government of Rwanda through the Ministry of Health created a public ambulance service called the Service d’Aide Médicale Urgence (SAMU).^20^ This service was created to provide timely prehospital care and to strengthen the health system.^21^ SAMU has grown from a Kigali-based ambulance service to being a country-wide service covered by community-based health insurance.^21^ More than 300 ambulances are deployed across the country, linked by a national dispatch centre and a free emergency service number (912).^22^ SAMU transfers approximately 8000 emergency patients per year, around 70% of whom have injuries.^12 23^ The remaining 30% have emergency medical or obstetric conditions.^23^ Prehospital care services are provided by ambulance crews, and a data-based quality improvement programme was put in place to improve the quality of prehospital care.^24^

**Figure 1:**
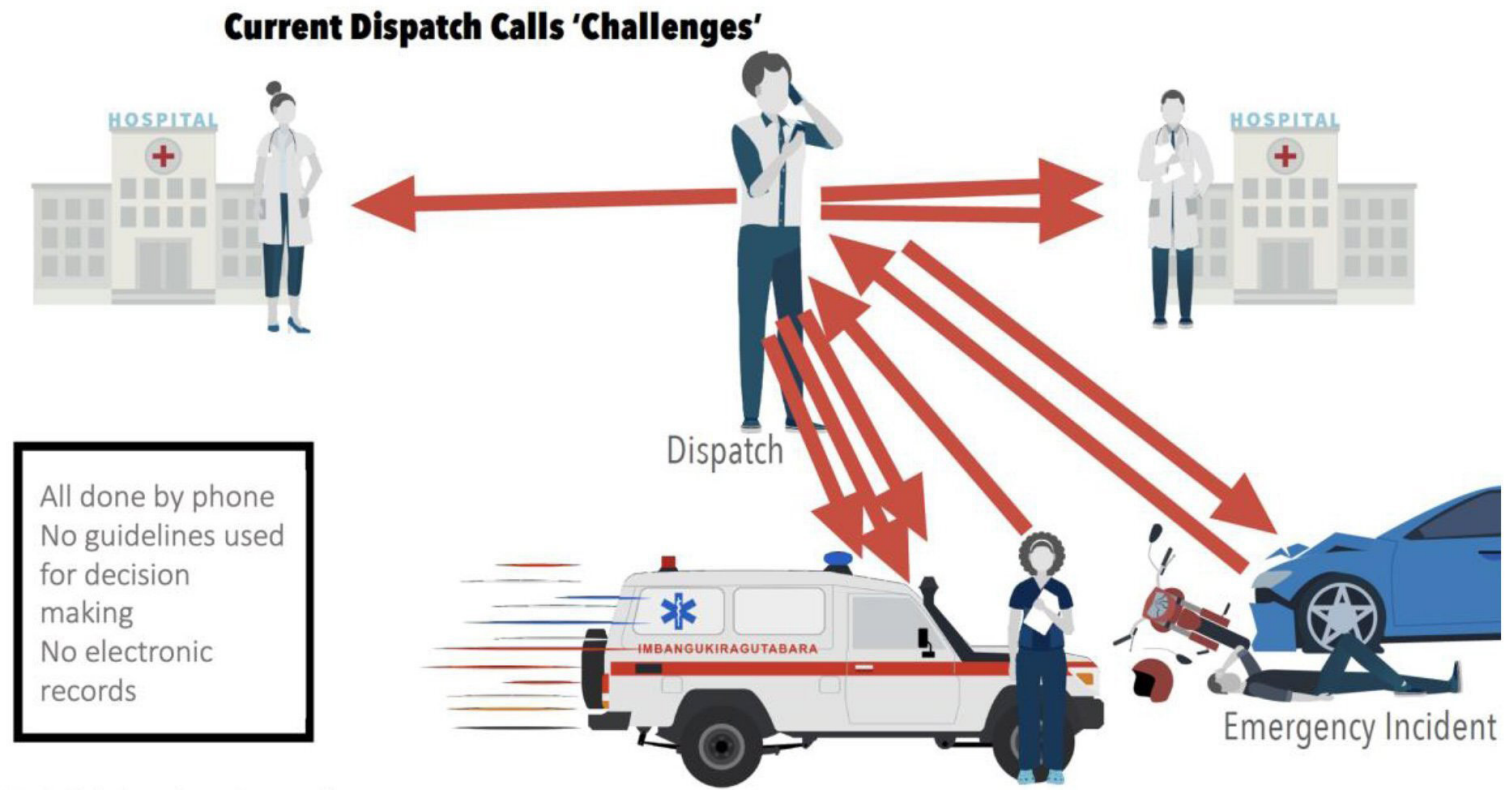
Inefficient communication (red arrows denote multiple mobile telephone calls) among the dispatch centre, ambulance crews and health facilities, resulting in avoidable prehospital delays. Note: Each red arrow is a phone call.

Similar to other ambulance services in African countries, all prehospital communications are done using mobile telephones.^7^ We have identified that inefficient mobile telephone communication and coordination between ambulance, dispatch and receiving hospitals (figure 1), combined with a limited use of triage guidelines, results in an average journey time of 1 hour 15 min to reach hospitals in Kigali (SAMU unpublished data, 2023). This represents up to 30 min of additional time from the set SAMU target journey time (45 min) from emergency location to hospital, with 42% of trips taking longer than 1 hour (SAMU unpublished data, 2023).

Additionally, there are multiple potential health facilities to which emergency patients can be transported. However, ambulance crews have no formal guidelines to triage patients to the right facility for them. Although the reasons have not been formally assessed, 22% of all SAMU journeys are interfacility transfers (unpublished data), which potentially reflects a substantial number of patients for whom the initial facility selected did not have all the requirements to deal with their condition.

Reducing delays in patient care arising from inefficient prehospital communication requires intersectoral collaboration and, in the setting, is best achieved through innovative, science-based, simple, low-cost, locally developed and locally supported solutions. Building on formative work bringing together industry professionals, academics and policy actors, 912Rwanda, a bespoke platform, was developed by the Rwanda Build Program, a local software company and implemented by SAMU in 2023. The goal of this project is to build on that platform and add the destination decision support algorithm (DDSA) capabilities and further improve the efficiency of the prehospital care system by connecting patients to the nearest ready hospital to treat them. Online supplemental appendix 1 describes the theory of change of this project.

### Aim

Our aim is to develop, roll-out and evaluate the effectiveness of a DDSA added to the existing electronic communication platform ‘912Rwanda’, which will link patients requiring prehospital ambulance services to the closest facility that is ready and able to treat their condition(s). In this study, we focus on the development, training and roll-out (study objectives 1–3). The second element, evaluation (objective 4), will be described in another sister study.

## Methods

### Setting

Prehospital care and emergency services are being expanded in Rwanda and our descriptions represent the situation at the start of the project.

This work will be conducted in SAMU and health facilities in Kigali city and Musanze district in Rwanda. Kigali was selected for study given it is the largest conurbation in and capital city of Rwanda. Musanze district was selected as a rural site; although the district hosts the second largest city in Rwanda, the patient population catchment is predominantly rural.

In Kigali, there are five district hospitals (Kibagabaga, Masaka, Muhima, Nyarugenge and Kacyiru) and three referral hospitals (Centre Hospitalier Universitaire de Kigali, King Faisal Hospital and Rwanda Military Referral and Teaching Hospital). These hospitals receive emergency and trauma patients from the local area, many of whom are transported by SAMU ambulances.^15^ The 36 health centres in Kigali receive less urgent cases.

Ruhengeri Referral Hospital (RRH) is the main referral hospital in Musanze district. This hospital receives emergency patients from the local area, mostly via requests from health centres (primary health facilities), rather than from direct patient or bystander requests. Emergency patients are stabilised in RRH before being referred to Kigali if deemed necessary. Given the lack of ambulance services to transport patients from the scene of the emergency and that most emergency patients present initially to health centres, the identified need from our preliminary research is for a system to triage patient transfers from health centres to RRH. In Musanze, there are usually five available ambulances; at any one time, two are generally occupied with transferring patients to Kigali. There is a dedicated team of seven nurses who oversee prehospital care.

### General description of 912Rwanda

The intervention, 912Rwanda, is designed and developed in two phases. In phase I, which is now complete, the interfaces in the dispatch centre, ambulances and receiving health facilities and a software platform were developed, tested and implemented. Phase I developed the foundations of 912Rwanda, including a web interface in dispatch to enter caller data, a map-based system to locate the patient, a mobile interface in ambulances to receive details of incidents and their location and a process to send SMS messages to facilities to alert them as to incoming patients.^25 26^ This phase is funded by the National Institute of Health (NIH—1R21TW011636-01A1) Fogarty Grant and an implementation report is in progress.

Phase II involves the development, testing, implementation and evaluation of the DDSA. This protocol describes phase II, which builds on the first phase^25 26^ and aims to develop a DDSA that uses information on facility and patient location (from phase I), the status of the patient (collected at the scene by SAMU staff) and the readiness of facilities to treat conditions (entered at health facilities).

### Patient and public involvement

Patients and/or the public were involved in the design of the project. A community engagement group (injured persons community group) was formed and will be involved in the conduct, reporting and dissemination plans of this research. The group participants gave written informed consent prior to their participation.

#### DDSA inputs

**Figure 2:**
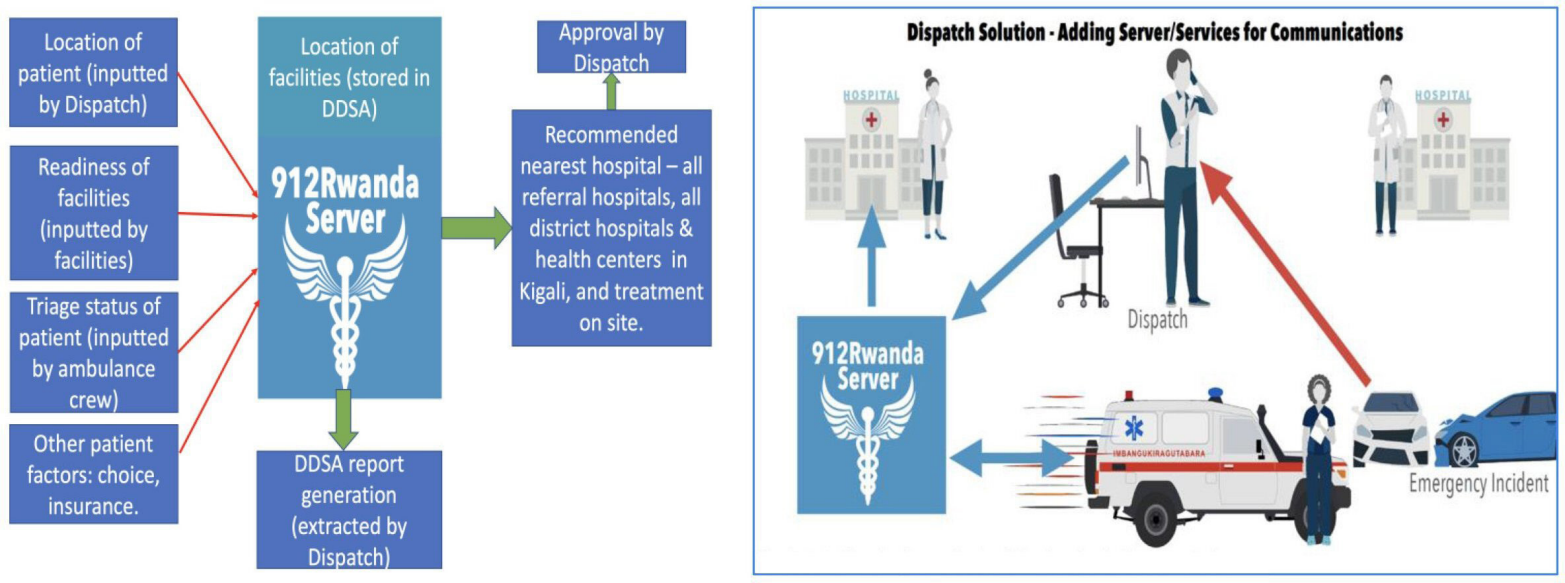
The destination decision support algorithm (DDSA) concept and solution. Note: Each red arrow is a phone call and each blue arrow is mHealth communication.

The DDSA will receive inputs from three main sources, namely, ambulance, dispatch centre and health facilities (figure 2). The users of the system will enter routine data into interfaces. All data will be used for SAMU reporting and operations. A subset of these data will be fed through the DDSA as follows: dispatch enters details of the location of the emergency, the patient’s gender and reported condition into a web interface in dispatch. This is sent to a mobile interface held by ambulance teams (this element has been developed, pilot tested and rolled out in phase I^24^). At the scene, ambulance staff will input information on the clinical status of the patient and collect other patient-related factors relevant to the destination decision.^27^ Dynamic facility readiness to receive patients (eg, availability of staff, beds, equipment and essential treatments (such as blood, anaesthetics)) will be inputted by the facility staff at least twice daily. Additionally, static facility readiness (the usual readiness of facilities to treat different types of emergency patients) and the location of facilities are held in the DDSA.

The DDSA is expected to send an alert to the ambulance teams and/or dispatch of the selected facility and the rationale for this after receiving all inputs. This decision is either approved or over-ridden. If approved, the ambulance proceeds to the facility. If over-ridden, the ambulance team and/or dispatch manually enter their choice of facility and their rationale for this by means of a prepopulated survey form. This approval step, serving as a human ‘check’ on otherwise automated decisions, is standard practice in automated systems and is intended to mitigate errors such as those seen in the introduction of fully automated ambulance software in London or the recent issues seen in the airline industry with Boeing 737 MAX.^28 29^

### Development, training and roll-out research methodologies

**Figure 3:**
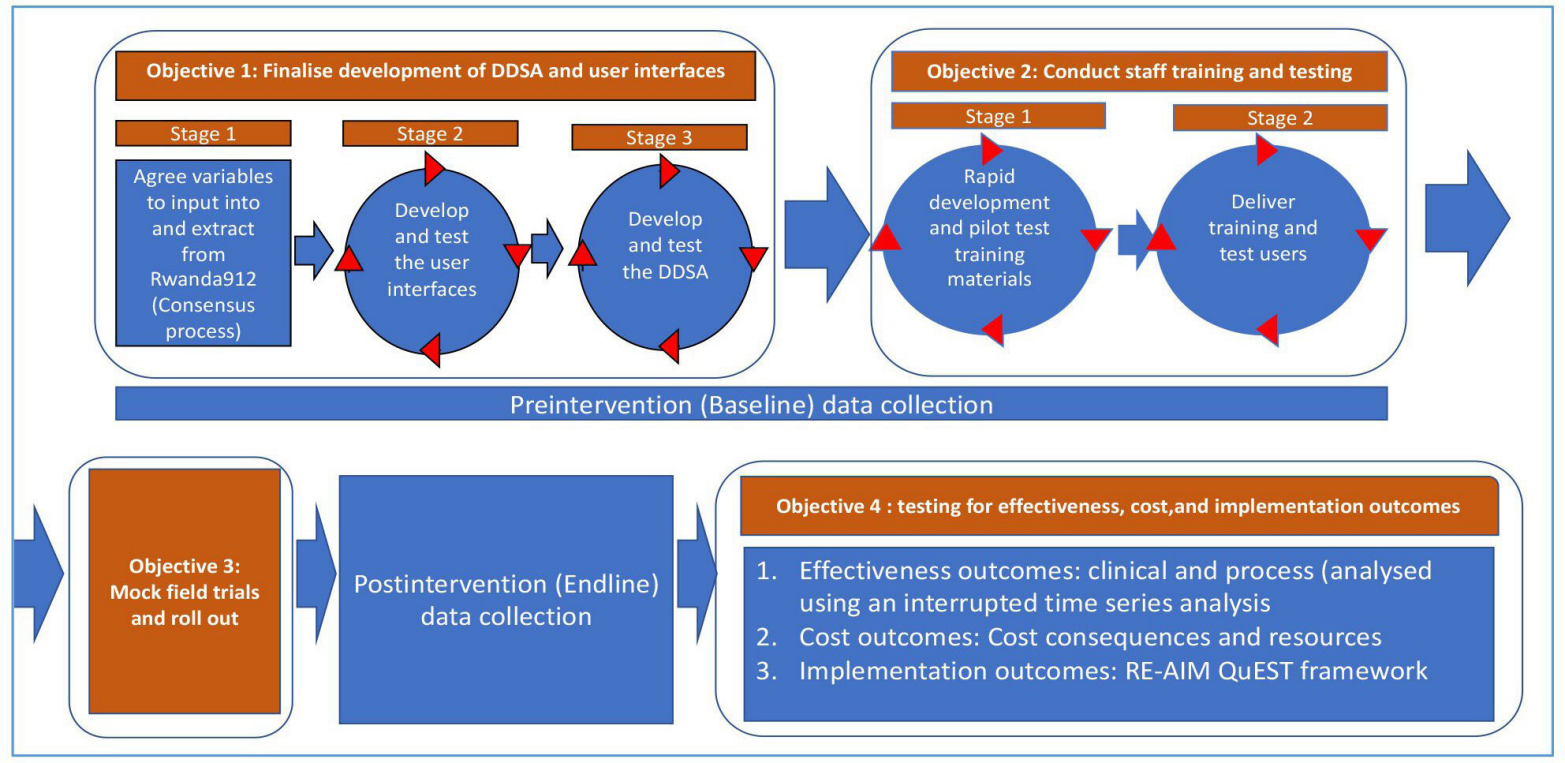
Summary of study flow. DDSA, destination decision support algorithm. RE-AIM, Reach, Effectiveness, Adoption, Implementation, and Maintenance; QuEST, Qualitative Evaluation for Systematic Translation.

The flow of the three main stages (each represented as an objective) of the development phase is presented in figure 3. Online supplemental appendix 2 shows the proposed study timeline. Analyses of the processes will be done to maximise the potential to transfer results to other settings.

#### Objective 1: develop 912Rwanda’s DDSA and user interfaces

**Figure 4:**
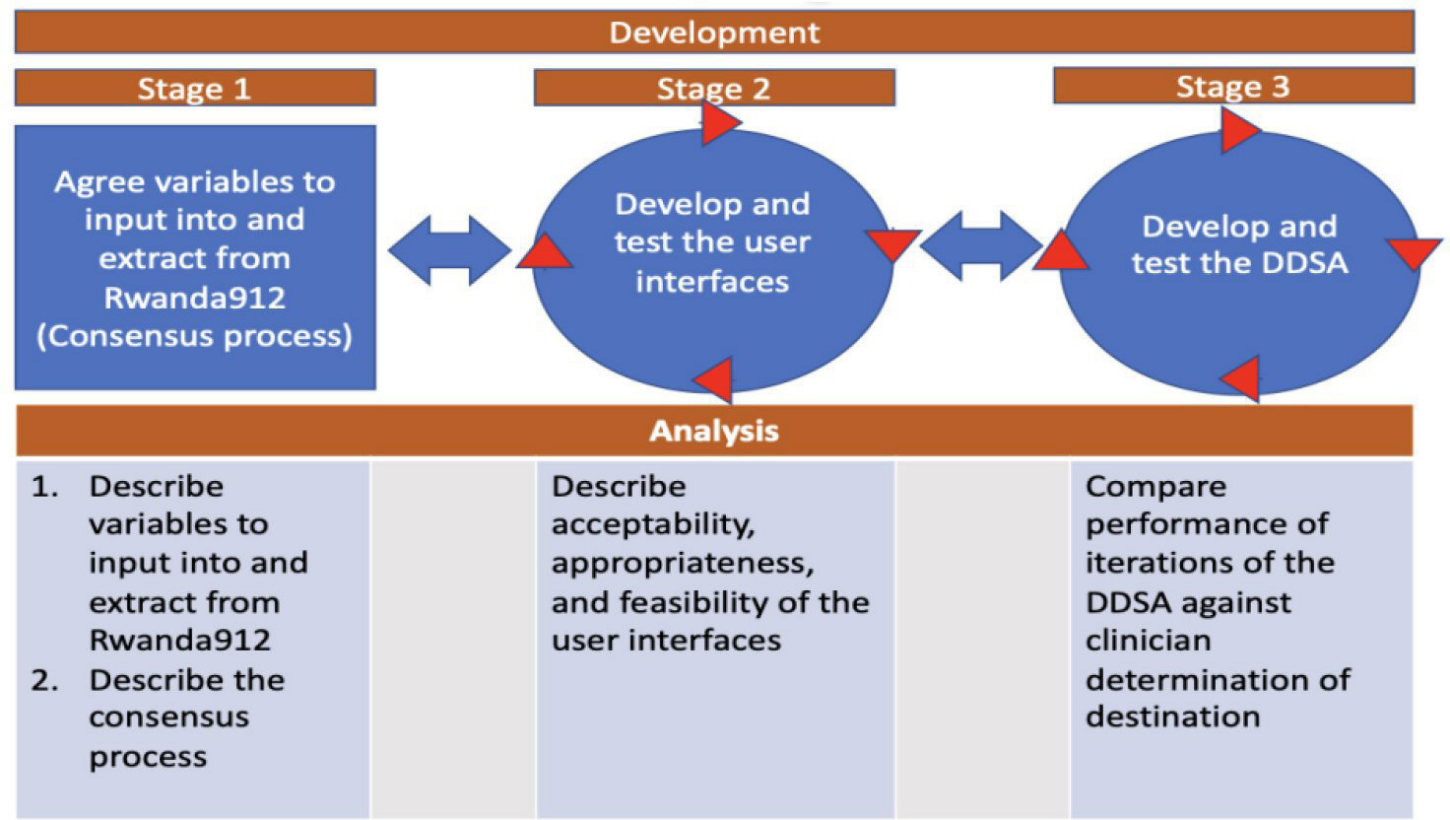
Objective 1: stages and analysis. DDSA, destination decision support algorithm.

The first objective will be conducted in three stages: (1.1) to agree on variables to input into and extract from the 912Rwanda system, (1.2) to develop and test the user interfaces and (1.3) to develop and test the DDSA (figure 4). Objective 1 stages will be iterative, and there will be some overlap with potentially>1 stage being developed and tested at the same time.

To agree on variables to input into and extract from 912Rwanda, we will first appraise current data collection and use cases for emergency patients at SAMU (methods are described in online supplemental appendix 3). Findings will be presented at individual and multistakeholder workshops prior to discussions to gain consensus on variables which the DDSA will use.

User interfaces to input and receive information in ambulances and facilities will be developed and tested using human–computer interface (HCI) design principles through requirement analysis, designs and prototyping, as well as evaluation.^30^ Based on findings from the ‘variable workshops’, and with input from experts in emergency medical data capture, user interfaces will be developed in consultation with the nominal lead of the relevant user group (SAMU ambulance staff, SAMU dispatch staff, facility staff and Ministry of Health of Rwanda/Rwanda Biomedical Centre (MoH/RBC)) and the research team leads. The interface prototypes will be iteratively developed until no further user improvement can be identified (methods are described in online supplemental appendix 3).

The algorithms that form the DDSA have potentially varying degrees of complexity. An appropriate method will be chosen from among decision trees (derived from basic manual or advanced machine learning), Bayesian networks and a hybrid approach (decision tree+Bayesian network). The configuration complexity chosen will depend on what is currently used for decision-making by SAMU and the amount of current data available to train and test an algorithm. Figure 2 illustrates the DDSA solution and concept (methods are described in online supplemental appendix 3).

#### Objective 1: analyses

Figure 4 describes the main analyses that will be done for each stage of the development in objective 1. In particular, the analysis for each stage will be done as follows.

##### Agreeing variables

Priority lists and consensus outputs will be described using the terminology given by the group participants, with language adjusted when necessary for clarity. The records taken during the sessions will be used where clarity is needed in preparing the consensus outputs.

##### Meeting notes analysis

Field notes on workshop proceedings will be analysed qualitatively using thematic analysis with particular attention being given to the rapidity and challenges of developing consensus, participant interactions, facilitators and barriers to the development of consensus and the impacts of hierarchy on discussions.

##### User interfaces and DDSA testing

User interfaces of each iteratively developed prototype will be tested using a collaborative heuristic approach^31^ using three methodologies: scenarios and personas, prototype testing using HCI principles and expert evaluation. Interface testing will focus on the way that data are entered (whether medical/data capture hierarchies and flow of data capture are logical) and the usability of the interfaces to capture data. A mixed-methods research evaluation and tools will be used. Data capture tools will include think-aloud sessions and focus group discussions and usability survey tools.

The DDSA prototype’s destination decisions will be tested against pre-existing decisions made by SAMU (using existing SAMU databases detailing patients and destinations) and by clinical experts; iterations of the DDSA will be developed to accommodate identified errors. Iterations will be continued until decisions made by the DDSA are 90% accurate to historical and expert destination decisions. Agreed thresholds to judge usability/safety/success of the DDSA and its interfaces from discussions will be described and applied in the analysis.

### Objective 2: develop training materials and conduct staff training and testing in a classroom setting

**Figure 5:**
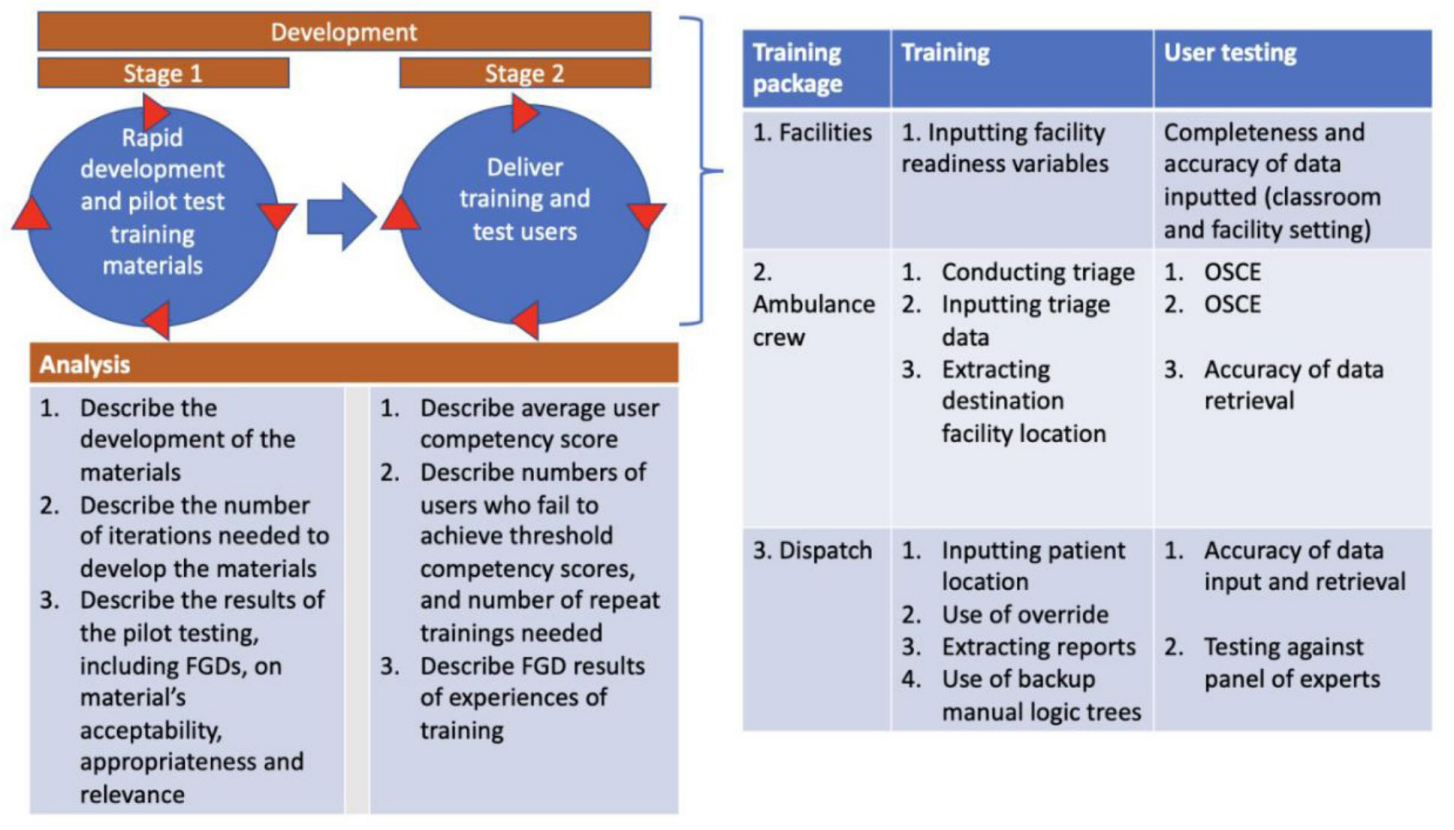
Objective 2: stages and analysis. OSCE, objective structured clinical examination. FGD, Focus group discussion.

The second objective will proceed in two stages: (2.1) rapid development of training materials and (2.2) delivery of training and user testing (figure 5). Training and testing materials will be developed after interfaces have reached their pre-final stage. The training materials will consist of a training booklet (including standard operating procedures for each user interface), lecture materials and quick guidelines. These materials will be piloted with users of each interface (dispatch, ambulance, health facility) using mock scenarios, with iterations of the materials developed to address issues discovered during focus group discussions with users. See online supplemental appendix 4 for details.

### Objective 2: analyses

Figure 5 describes the main analyses that will be done for each stage of the development in objective 2. A computer simulation programme for training and testing using mock scenarios for ambulance crews will be developed. Subsequently, training will be delivered, and user competency assessed with all users until the target competency of ≥90% on completeness and accuracy of data inputted to their relevant interface is achieved. We will run the objective structured clinical examination for SAMU ambulance crews for inputting triage data and for facility staff inputting readiness data. Online supplemental appendix 4 describes the methodologies for objective 2 in full.

#### Iterative development

The first and second objectives are conceived to be iterative, with stages/cycles of development, testing and approvals repeating until the interfaces and DDSA are considered acceptable at predetermined thresholds.

### Objectives 3: conduct mock field trials and roll-out the intervention

The third objective tests the entire product and will proceed in three stages: (3.1) sandbox scenario testing, (3.2) mock trauma scenarios and (3.3) roll-out.

In stage 3.1, the usage of the complete active platform will be tested by all users in classroom/computer-based simulated scenarios based on data from real patients from the Rwanda Trauma Registry and SAMU historical data. For this, the system will be operational in a test server/‘sandbox’. Approximately 200 scenarios will be tested over 1 month to ensure that all users have a chance to experience the complete system. Testing each scenario will require simultaneous testing with members from facilities, dispatch and ambulance crews.

In stage 3.2, mock trauma scenarios will be based on real patients from the trauma registry (and/or primary trauma care guidelines) and use dummy patients (actors) at different locations in Kigali. This stage’s aims are twofold: to test user competency and to test software reliability/functioning in the ‘real world’. All system users participating in the scenarios will enter and extract information in as close an approximation to the real world as possible. However, ambulance crews will not remove the ‘patient’ from the scene, and ‘ambulances’ may be replaced with taxis. Observers will be stationed at each ‘patient’, in each ‘ambulance’, in dispatch, and in facilities to record observations on user interaction with the software and the times that data are entered/received/extracted. Scenarios will run over 1–2 weeks and aim to involve all individual users of the system.

After these assessments are judged successful, as assessed by user competency and system reliability testing, the phase two software will be fully rolled out (stage 3.3). Roll-out will be done over 1 month, with the current system being replaced with the new system and all study investigators being on hand to discuss and resolve any problems. All issues and solutions will be recorded and reported. Of note, roll-out in Kigali will precede roll-out in Musanze. Online supplemental appendix 5 describes the methodologies for objective 3 in full. Online supplemental appendix 6 outlines the overarching qualitative methodologies that will be employed throughout the course of the study.

## Discussion

This health system strengthening project aims to develop, test and integrate a unique, locally developed DDSA into a bespoke emergency medical services communication system based on specific features, needs and circumstances of the Rwandan prehospital care system. A collaborative approach informed by the leadership of MOH/RBC and local experts through deep engagement of key stakeholders will be used. The project holds great promise for improving the efficiency of emergency medical services and ultimately saving lives. To achieve this, we will work to embed 912Rwanda into policy by close engagement with all relevant stakeholder groups. Additionally, we will collaborate with multilateral policymakers from study inception to share our results and promote global uptake. Patient and public participation is a crucial aspect of our study, and our community engagement and involvement strategy ensures active involvement throughout the project—from design to dissemination. Our goal is to foster sustainable engagement in translating research into effective policies.

The capture of the development process using rigorous methodologies will produce findings which are likely to improve ambulance software development processes, including increased efficiency and quality of software products, in similar LMICs settings. The project involves multisectoral collaboration between academia and industry will foster a deep understanding of practical challenges and facilitate the integration of theoretical and practical knowledge.

Our study has limitations, the main of which is the likely lack of availability of high-quality data in Rwanda to understand the current system and facilitate the development of the DDSA. While both SAMU and individual healthcare facilities currently collect numerous data points on emergency cases, the completeness and quality of these data may be insufficient to support a data-based approach. We have therefore developed our methods throughout the programme to use as a common foundation and situated expert practitioner opinion to mitigate against data that may be insufficient, incomplete or unavailable. Expert opinion approaches rely on input from providers in a low-resourced setting, and we recognise that their capacity to participate may be challenged. Nevertheless, from our experience of phase I, there is broad enthusiasm from collaborators across multiple sectors of the prehospital care system in Rwanda for this solution; together with a willingness to provide the time and resource to ensure it is developed with a high degree of fidelity and integrity for, and with the local context. Finally, we acknowledge that the field of software development is dynamic, with rapidly evolving technologies, particularly in the machine learning space. As a result, research projects and findings may become outdated relatively fast, making it essential to stay updated with the latest developments.

## Ethics and dissemination

The project, as well as all consent forms and research tools, has been approved by the Rwanda National Research Ethics Committee (Ref No: 99/RNEC/2023). Approval has also been sought from the hospital authorities to access physical and electronic medical records. Anonymised healthcare data from patients will be used and patient consent will not be required. Informed consent will be obtained from healthcare providers and policymakers prior to participation in this study. All methods in this study will be carried out in accordance with relevant guidelines and regulations in the ethical declarations.

We will leverage established collaborations with key stakeholders—including the MoH, and their technical arm (the RBC), the national ambulance service (SAMU, Division of EMS and Dispatch), the software development team, healthcare providers and project partners who have been actively involved in both the design and implementation phases—to disseminate findings effectively to target audiences and maximise impact. Annual reports will be distributed to all stakeholders and subsequently made available to the public. Tailored presentation workshops will be offered to individuals responsible for integrating findings into practice. Additionally, policy briefs will be developed for members of the scientific advisory group to support broader dissemination to funders and wider audiences. In accordance with standard academic practice, study findings will be presented at scientific conferences and published in high-impact, open-access international journals. All methodologies will be made publicly accessible via the project website to facilitate replication and further research.

## Supplementary material

10.1136/bmjopen-2025-102355 online supplemental file 1

10.1136/bmjopen-2025-102355 online supplemental file 2

10.1136/bmjopen-2025-102355 online supplemental file 3
