# Supplementary figures and images for: Developing and integrating a destination decision support algorithm into an innovative electronic communication platform to improve injury care service coordination in Rwanda: the Rwanda912 study protocol
Source: BMJ Open. 2025 Jun 27;15(6):e102355. doi: 10.1136/bmjopen-2025-102355 (PMC12207105; doi:10.1136/bmjopen-2025-102355)

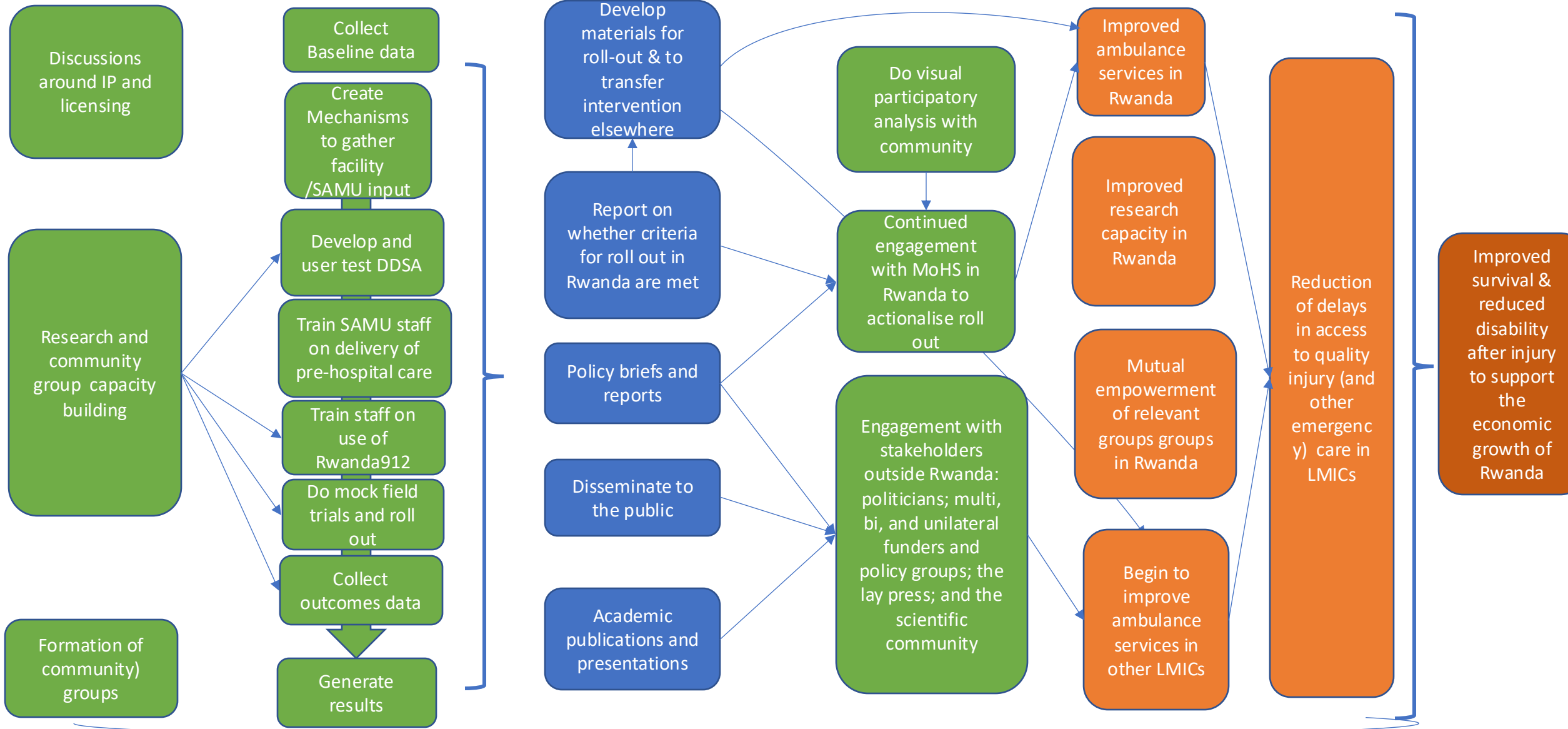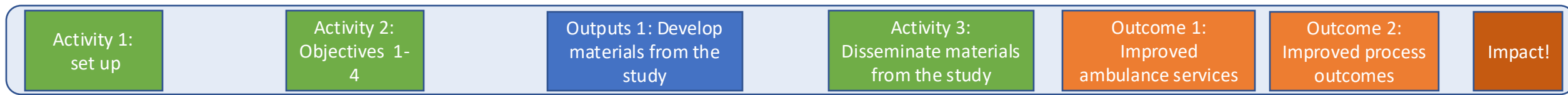

Supplement: online supplemental file 1 [file bmjopen-15-6-s001.pdf]
