# Supplementary material for: Developing and integrating a destination decision support algorithm into an innovative electronic communication platform to improve injury care service coordination in Rwanda: the Rwanda912 study protocol
Source: BMJ Open. 2025 Jun 27;15(6):e102355. doi: 10.1136/bmjopen-2025-102355 (PMC12207105; doi:10.1136/bmjopen-2025-102355)
Supplement: online supplemental file 2 [file bmjopen-15-6-s002.pdf]

Appendix 2_Gantt chart

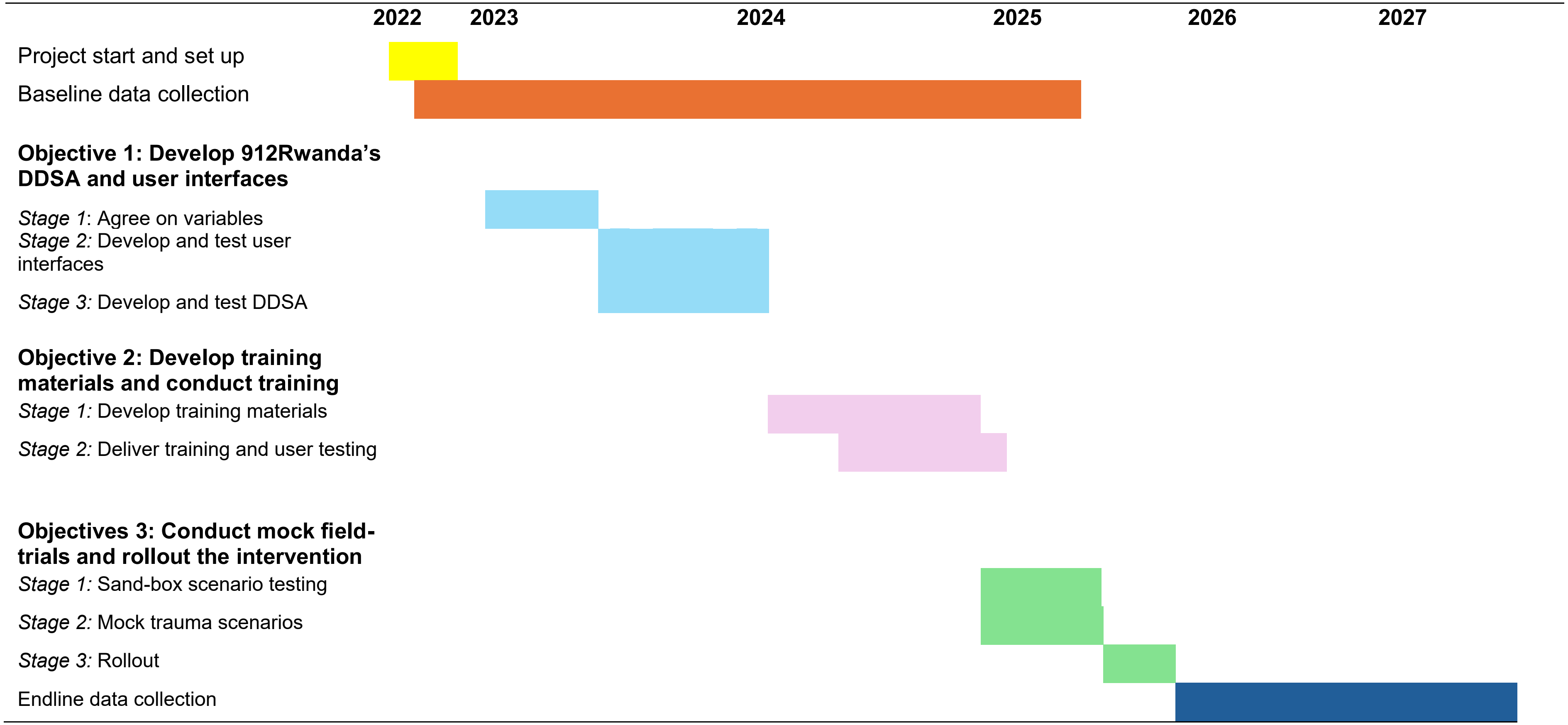

Figure: Project Gantt chart. Yellow represents project start and set up, orange represents baseline data collection period with no intervention, light blue represents objective 1, violet represents objective 2, green represents objective 3, and dark blue represents endline data collection after intervention.
