# Supplementary material for: Developing and integrating a destination decision support algorithm into an innovative electronic communication platform to improve injury care service coordination in Rwanda: the Rwanda912 study protocol
Source: BMJ Open. 2025 Jun 27;15(6):e102355. doi: 10.1136/bmjopen-2025-102355 (PMC12207105; doi:10.1136/bmjopen-2025-102355)
Supplement: online supplemental file 3 [file bmjopen-15-6-s003.docx]

**APPENDICES 3 – 6: DETAILED METHODOLOGIES**

Table of Contents

[**Appendix 3: Objective 1 Finalize development of Rwanda912’s Ambulance Destination Decision Support Algorithm (DDSA) and user interfaces.** 2](#_Toc182476361)

[**Objective 1, stage 1 – Agree variables to input into and extract from Rwanda912.** 2](#_Toc182476362)

[Methodology 2](#_Toc182476363)

[Understanding process and information pathways. 2](#_Toc182476364)

[Stakeholder workshops to agree variables to input into and extract from the Rwanda912 system. 4](#_Toc182476365)

[Specific workshop methodologies 5](#_Toc182476366)

[*Dispatch decision to override* 5](#_Toc182476367)

[*Field triage status and ambulance variables* 5](#_Toc182476368)

[*Patient factors* 6](#_Toc182476369)

[*Facility readiness and facility alert variables* 7](#_Toc182476370)

[*Reporting dashboards* 8](#_Toc182476371)

[*Co-design workshop* 9](#_Toc182476372)

[Overall summary 9](#_Toc182476373)

[**Objective 1, stage 2 – User interface development and testing** 9](#_Toc182476374)

[Summary 9](#_Toc182476375)

[Development 9](#_Toc182476376)

[UIx testing 9](#_Toc182476377)

[*User interfaces to be tested* 10](#_Toc182476378)

[*User Participants* 10](#_Toc182476379)

[Methods 10](#_Toc182476380)

[1. *Prototype user interface testing* 10](#_Toc182476381)

[*2. Summative evaluation* 12](#_Toc182476382)

[3. Testing of pre-final interfaces by all users 12](#_Toc182476383)

[**Objective 1, stage 3. Algorithm development** 13](#_Toc182476384)

[KPIs 13](#_Toc182476385)

[Iterative development 14](#_Toc182476386)

[System Risk testing 14](#_Toc182476387)

[**Appendix 4: Objective 2 – Develop training materials and conduct staff training and testing in a classroom setting.** 14](#_Toc182476388)

[**Objective 2, stage 1: Development of training materials** 15](#_Toc182476389)

[Summary 15](#_Toc182476390)

[Methods for development and pilot testing of training materials 15](#_Toc182476391)

[**Objective 2, stage 2: Delivery of training and testing user competencies** 16](#_Toc182476392)

[Training delivery 16](#_Toc182476393)

[Assessments of delivery of and utility of training 16](#_Toc182476394)

[Testing user competency 16](#_Toc182476395)

[Development of material to be used in user competency testing 16](#_Toc182476396)

[**Appendix 5: Objective 3 - Conduct mock field-trials and roll out of the intervention** 18](#_Toc182476397)

[**Objective 3 stage 1: Sand-box scenario testing** 18](#_Toc182476398)

[**Objective 3 stage 2 - Mock Trauma Scenarios** 19](#_Toc182476399)

[Roll out 21](#_Toc182476400)

[**Appendix 6: General Qualitative methodologies** 21](#_Toc182476401)

[Summary 21](#_Toc182476402)

# **Appendix 3: Objective 1 Finalize development of Rwanda912’s Ambulance Destination Decision Support Algorithm (DDSA) and user interfaces.**

Objective 1 will be conducted in 3 stages. These stages will be iterative and there will be some overlap with potentially >1 stage being developed and tested at the same time.

# **Objective 1, stage 1 – Agree variables to input into and extract from Rwanda912.**

## Methodology

## *Understanding process and information pathways.*

Kigali

*Information pathways* mapping.

To capture which information is used to make decisions, where that information originates from, and how it is used, we will do information pathway mapping. This will be done in a workshop with participants together from ambulance dispatch, driver, and facility teams (ideally ~3-4 from each group to a total of 9-12).

Contextual group interviews were done in the development phase of the NIH grant and used in development of an initial process map (figure 1). Participants will be presented with the process map – below – to check its correctness, and if not correct, make changes in real time. Participants will then be asked to list the information that they use at each decision question to enable a decision to be made. The research team will capture this information as a list for each stage.

At the end of the session, respondents will have the project described to them in summary and be presented with a graphic of the proposed new system. They will also be requested to consider, for future meetings (detailed below), in the new system, what variables that each group (ambulance crew, dispatch, and facility) would find vital or desirable to collect or see in the electronic interfaces, and for each of these categories, which they feel are feasible to input or assess.


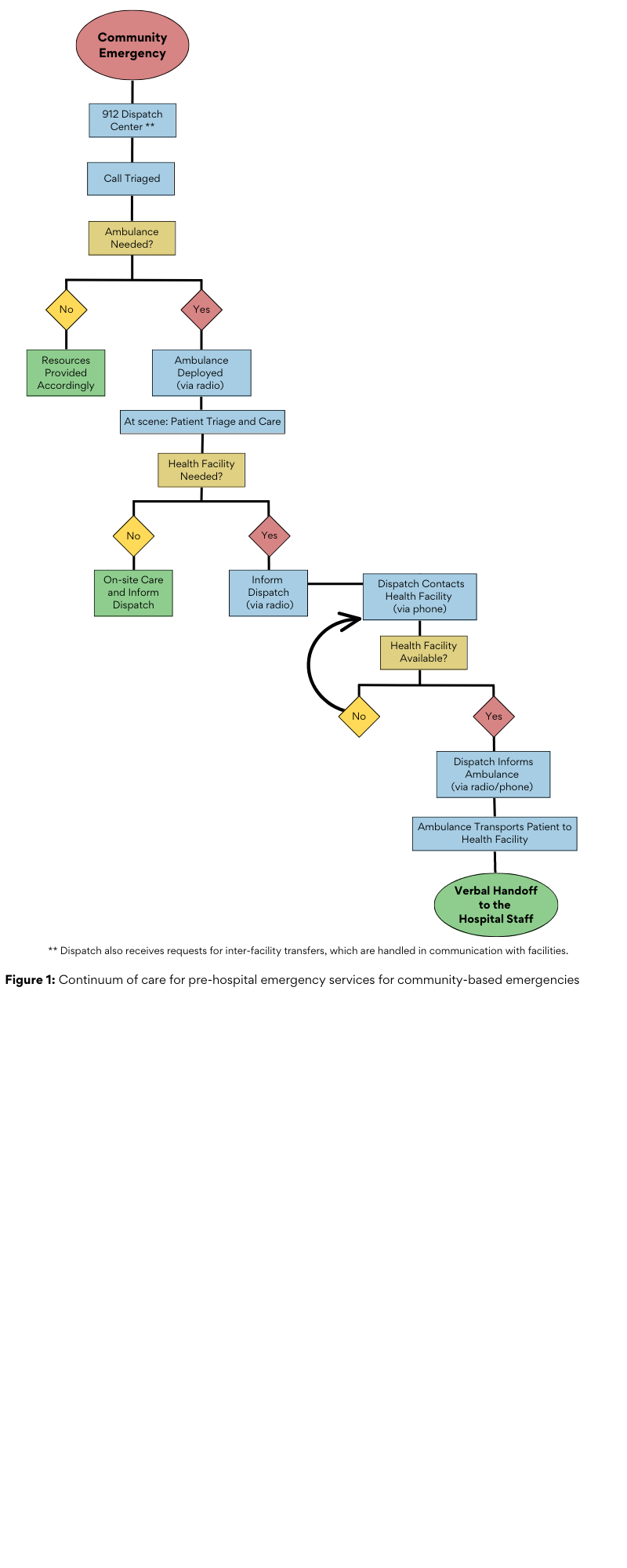


Musanze

No prior process or information flow mapping has been done in Musanze. Here, contextual group interviews will be carried out with members of the ambulance dispatch, driver, and facility teams (ideally ~3 participants from each group). These will capture the work/tasks of each group, and the problems experienced. During this process a facilitator will create a real time process and information flow map under the direction of participants.

Once the group are happy with their map the researcher/facilitator will ask the group to describe and discuss issues that they currently experience as they move through the process and what could facilitate these processes. These are noted.

At the end of the session, the outputs are captured to produce summaries for recirculation amongst group members for further input/agreement.

On a second occasion – once the process map is agreed - the group should be reconvened to produce information pathway maps – done as per Kigali.

## *Stakeholder workshops to agree variables to input into and extract from the Rwanda912 system.*

At the end of the process mapping, described above, respondents will have the project described to them in summary and be presented with a graphic of the proposed new system. They will be requested to consider, for future meetings, in the new system, what variables that each group (ambulance crew, dispatch, and facility) would find vital or desirable to collect or see in the electronic interfaces, and for each of these categories, which they feel are feasible to input or assess. They will be invited to attend stakeholder workshops, described below.

Note that data on facility location, facility designation (private, public, semi-private, etc, and whether facility accepts people who are uninsured/have community health insurance) will be held as static information in the DDSA. Patient location, as collected by phase 1 of 912Rwanda, will be included in the DDSA.

Format of the workshops

Workshops will take half to one day, depending on the issues under discussion. We will utilise a nominal group process to develop consensus at workshops. Discussions will be held in plenary to describe the nature and aims of the workshops. Following, small group/roundtables will be convened to discuss particular issues (e.g: variables to enter each interface) and develop consensus. Each small group will present their consensus in turn in plenary with a facilitated discussion to explore differences and rationale between groups. Consensus will either be developed in the group discussions or individuals will use voting to achieve consensus; methodologies will be determined by the degree of consensus achieved after discussion and the issue under consideration.

Each small group/roundtable discussion will be facilitated by a study team member with facilitation being sensitive to the potential hierarchies in each group and done to ensure maximum contribution to discussions from all attendees.

Data capture

Lists or summaries of outputs will be captured by the facilitator.

Ethnographic data on the proceedings of the meetings and the process of coming to consensus will be captured by a trained observer taking anonymised field notes covering issues of key points of discussion, speed of reaching consensus, agreement, and disagreement, and equitability of input from all stakeholder groups.

Analysis

Outputs will be described using the terminology given by the group participants, with language adjusted when necessary for clarity.

*Meeting process*:

Field notes on meeting proceedings will be analysed thematically (see qualitative methods, below) with particular attention being given to the rapidity and challenges of developing consensus, participant interactions, facilitators and barriers to development of consensus, and the impacts of hierarchy on discussions.

### Specific workshop methodologies

### *Dispatch decision to override*

Method

A half to full day workshop attendees to determine factors to trigger dispatch override of the DDSA.

Attendees

1. *SAMU staff*, including dispatch and ambulance crew, including Musanze (Other outside Kigali) (<8 pax)

Factors to be considered

1. Discussions on need for dispatch over-ride
2. Agreement on need for dispatch override
3. Discussion on how dispatch would like to see outputs and recommendations from DDSA e.g:
   - simply the hospital,
   - the hospital with a probability match
   - the hospital with reasons why selected
   - the hospital with a dashboard of indicators showing factors that matched and those that didn’t (if so – which indicators should be shown)
4. Discussion on the feasibility of interpreting in real time the above discussed options
5. Agreement on the ideal and reasonable way of seeing outputs
6. Discussion of variables to be entered to justify dispatch override. E.g.:
   - more up to date knowledge of facility readiness (and why?)
   - knowledge of other emergencies sent to chosen facility
   - knowledge of traffic delays en-route to selected facility
7. Agreement on variables to enter to justify over-ride
8. Discussion on user acceptability of interfaces and safety thresholds DDSA to exceed before deployment

### *Field triage status and ambulance variables*

Preliminary discussions have suggested that the current data captured in the triage process should be considered along with potential modifications based on the United States Center for Disease Control (CDC) field triage guidelines.

Method

A half to full day workshop to determine variables to be captured by SAMU staff at the scene.

Attendees

1. *SAMU staff*, ambulance crews, including Musanze (<10 pax)

Factors to be considered

Discussions will consider input of field triage information, and receipt of destination decisions.

1. Presentation of current SAMU field triage/data capture processes
2. Presentation of other field triage guidelines (e.g. CDC Field Triage guidelines).
3. Discussions on feasibility of current field triage/data capture processes (including method of data collection and volume of data collected)
4. Discussions on necessity to change/add variables to current data collected
5. Listing of variables that are desirable to capture
6. Prioritising variables from the above list or variables that are vital to capture
7. Considering all listed variables – which are feasible to capture in real time in the field
8. Discussion of how to receive dispatch approved DDSA decision
9. Discussion on user acceptability of interfaces and safety thresholds DDSA to exceed before deployment

### *Patient factors*

Preliminary discussions have identified that patient factors, like choice of facility to be taken to and insurance status should be considered for capture.

Method

A half to full day workshop to determine which patient factor variables to be captured by SAMU staff at the scene

Attendees

1. *Service users* (community members) (<6 pax)
2. *SAMU staff*, including dispatch and ambulance crews, including Musanze (Other outside Kigali) (<6 pax)
3. *Facility staff*, including managers and emergency department clinicians, including Musanze (<5 pax)
4. *Policy makers*, including MoH and Rwanda Biomedical Centre (<4 pax)

An indicative guide for the workshop proceedings is presented as follows:

1. Presentation on the purposes of the project and how the patient preference variables are proposed to be inputted ambulance staff and used in the DDSA
2. Presentation of current patient considerations accounted for when deciding action to take and how these considerations affect decision making
3. Discussions on whether the currently used patient factors should continue to be captured
4. Discussions on any new patient factors that should be captured
5. Listing of all patient variables that should be captured
6. Listing of those that are vital and desirable
7. Noting of the feasibility of capturing each and all of the above list of variables n electronic means in real time at the scene.
8. Discussion on user acceptability of interfaces and safety thresholds DDSA to exceed before deployment

### *Facility readiness and facility alert variables*

- The facility will:
- Capture data on readiness and input this to the DDSA

Receive an alert (on the web interface/other) that the patient is arriving (and their characteristics)

- Potentially over-ride incoming patient decisions

Facility readiness variables

- Preliminary discussions have identified that these variables should be available already or will be readily collectable and are based on variables currently used to inform human-based decision-making about destination hospitals.
- These variables may include bed status and availability of ITU, staff, blood, working essential equipment. Many of which are already regularly collected manually onto paper.
- Readiness variables will include those for non-trauma emergency conditions, given that SAMU is sometimes used for other – especially obstetric – emergencies.

Frequency of input of facility data (e.g.: once, twice, or 3 times per day) will be agreed, as will the nominated persons to input these data at the facility.

- Variables the facility would like to see to help them to prepare for the patient need to be identified.

Method

A half to full day workshop to determine variables to be captured by facility staff to indicate readiness of facilities to receive patients.

Attendees

1. *Facility staff*, including managers and emergency department clinicians, including Musanze (<7 pax)
2. *SAMU staff*, predominantly dispatch, given they utilise these data currently to make decisions (including from Musanze)

An indicative guide for the discussion proceedings is presented as follows:

1. Presentation on the purposes of the project and how the facility readiness variables are proposed to be inputted by facility staff and used in the DDSA
2. Presentation of current data that facilities collect on readiness, the frequency of collection, by whom, how data are collected, and the usefulness or otherwise of current methods of data collection
3. Presentation of facility readiness variables used in similar decision-making in other countries, e.g. High- Income Country Emergency Ambulance Destination systems.
4. Presentation of current considerations used by Dispatch staff in determining destination facility readiness
5. Discussions on necessity to change/add to current data collected at facilities to match what dispatch need
6. Discussions to consider any additional variables that the facility think are important
7. Discussions on the feasibility of collecting the above additional variables
8. Discussions on feasibility of processes to capture data to enter into an electronic interface (how data are captured, by whom, and frequency of data capture)
9. Discussions on the facility alert – what information are facilities currently given on incoming emergency patients
10. Discussions on the facility alert – discuss and list alert variables to receive that are vital to have and nice to have, considering the feasibility of interpreting the information in the workspace
11. Discuss the ideal format of the facility alert
12. Discuss facility ability to override

- should this be a feature?

- if so what should it be based on?

- what data should be sent to justify the decision

1. Discussion on user acceptability of interfaces and safety thresholds DDSA to exceed before deployment

### *Reporting dashboards*

A simple dashboard will likely include all data inputted into the DDSA, transport time data collected by the DDSA, destination decisions made by the DDSA, and overrides by dispatch and the reasons for those overrides.

These data will be used for ongoing Quality Improvement (QI) purposes by SAMU and - for this current study - in assessing endpoints.

This workshop will occur after other workshops have agreed variables

Method

A full to half-day workshop to determine reporting dashboard format, content, data extraction, and use

Attendees

1. *SAMU staff*, including dispatch and ambulance crews, including Musanze (<6 pax)
2. *Facility staff*, including managers and emergency department clinicians, including Musanze (<6 pax)
3. *Policy makers*, including MoH and Rwanda Biomedical Centre

An indicative guide for the workshop proceedings is presented as follows:

1. Presentation on the purposes of the project and the variables to be inputted into and derived by the DDSA
2. Plenary presentation on the current uses of data for quality improvement by SAMU and facilities
3. Presentation on the uses of data for QI by external organisations
4. Discussions on which departments require data for QI and suggested uses of data
5. Discussions on variables/summary outputs to be displayed in dashboards for different departments
6. Listing of variables that are vital
7. Listing of variables that are desirable
8. Agreement of variables to output
9. Discussions on the format of variables/summary outputs
10. Agreement of formats that are ideal and reasonable
11. Discussions on frequency of reporting needed for different departments
12. Agreement on frequency of outputs that are ideal and reasonable
13. Discussion on user acceptability of interfaces and safety thresholds DDSA to exceed before deployment

### *Co-design workshop*

We will collate findings from the above workshops and present a summary of each of the variables to collect and the flow of variable collection for each user interface to RWBuild, SAMU, RBC, and facility leaders. We will solicit feedback on suggestions for flow from participants and adapt suggestions in real time.

We will utilize information to work with the software team to develop initial mockups of user interfaces. We will additionally agree threshold acceptability for user interfaces, the complete software, and training.

### Overall summary

During stakeholder workshops, we will gain agreement on

1. The list of variables to be inputted into Rwanda912’s DDSA.
2. The list of variables to be extracted from Rwanda912’s DDSA.
3. Acceptability and safety thresholds for Rwanda912 to progress to next phases of testing/roll out when appropriate.
4. The use of an over-ride by Dispatch

# **Objective 1, stage 2 – User interface development and testing**

## Summary

This stage is iterative, with stages/cycles of design, testing, provisional user leader approval, User Experience testing (UIx), redesign, testing, provisional leader approval, UIx, etc until the interfaces and data captured are considered acceptable at thresholds determined by users in the workshops (phase 1, above).

## Development

Based upon findings from workshops above, and with input from experts in emergency medical data capture, user interfaces will be developed in consultation with the nominal lead of the relevant user group (e.g.: SAMU ambulance staff, SAMU dispatch staff, facility staff, and MoH/RBC) and the research team leads for this objective.

## UIx testing

This objective will test both:

1. The way that data are entered (whether medical/data capture hierarchies and flow of data capture are logical)
2. The usability of the interfaces to capture data

### User interfaces to be tested

1. Field triage and patient factor interface
2. Facility input and output variables interface
3. Dispatch over-ride interface
4. Dashboards

### *User Participants*

Per session there will be between 3-5 of each user as below:

1. Field triage and patient factor interface

- SAMU ambulance staff

1. Facility input variables interface

- Facility staff who are nominated to collect these items (to be decided in the workshop)

1. Dispatch over-ride interface

- SAMU dispatch staff

1. Dashboards

- SAMU staff
- Facility staff
- MoH/RBC data scientists

## Methods

Methods for user experience testing of each interface will be identical and will proceed as below.

### 1. *Prototype user interface testing*

User interfaces of each iteratively developed prototype will be tested using < 5 participants and < 5 scenarios and personas for each round.

Scenarios and personas

Personas

Personas of the typical user will be drafted to understand the type of users who will be interacting with the system and what they will be using the system for (their tasks, actions, and processes). Personas will need to be created based on the discovery work done in the NIH grant and additional discussions with users for this study.

Scenarios

To test the interface, users will be asked to perform tasks based upon pre-developed scenarios particular to their user-interface requirements. These scenarios will be developed by the research team in conjunction with relevant leaders of SAMU/RBC/healthcare facilities.

Dependent on user interface to be tested, content of scenarios will be informed upon real world cases – for example, ambulance triage data capture scenarios will be developed using triage forms currently in use and turning these into patient case scenarios. Facility data will be captured based on data from daily reports currently produced. Additional information for variables agreed to be captured in the workshops, but not currently captured will be integrated into these scenarios.

Scenarios will be paper based.

Evaluation

Data capture and analysis

In addition to data captured as described in the methods below, data will be collected on the participant demographics (age, sex) and place of employment

1. Think aloud

Individual users will be given basic training on the features of dummy interfaces on appropriate devices and oriented to the think aloud methodology. They will then be asked to use the interface and comment on acceptability or challenges experienced whilst they are using it. Their comments will be captured by an observer noting which issues are experienced when using particular features.

The observer(s) will not offer help in using the interface.

Think aloud sessions will be recorded (anonymously) in case clarity is needed when writing up results, but there is no plan to transcribe and analyse these sessions.

Each session will take around 45 mins.

Each interface will be tested during a ½ to one-day period

*Data analysis*

The day of/following the think aloud session the observers will synthesise results of the think-aloud testing noting the following:

- all positive and negative issues enhancing or limiting the usability/functioning of the interfaces experienced by users, the total number of times each issue was experienced, and the number of users experiencing the issue
- all positive and negative issues affecting the understanding of/data capture using the medical/data capture hierarchies, the total number of times each issue was experienced, and the number of users experiencing the issue
- all cosmetic comments around the use of the software, but which do not limit its usability/functioning, the total number of times each cosmetic issue was experienced, and the number of users experiencing the issue

Synthesis will be based upon the Stanford Lightening Report Method [6].

1. NASA-TLX questionnaire

At the end of each group of tasks, participants will be asked to complete the NASA-TLX questionnaire

This contains questions on the following domains:

- Mental Demand

- Physical Demand

- Temporal Demand

- Performance

- Effort

- Frustration

Data capture will be onto paper (with the research team transferring the results to the electronical data capture tool immediately after completion).

Data analysis

Results will be summarised for each domain of task load.

1. Focus group discussions

Following think-aloud testing, and on the same day, users will form a focus group to discuss their experiences with the interface and any challenges and offer suggestions for improvement.

FGDs will be recorded for transcription, translation (if necessary) and analysis. Participants will be asked to not identify themselves during the recorded discussion.

*Data analysis*

Rapid analysis of the FGD will be done based on the Stanford Lightening Report technique to capture themes on usability, cosmetics, and suggestions for improvement in these domains.(36)

Use of results of think aloud and FGDs

Results of the think aloud and FGDs will be prepared as a brief report and discussed with the software developer team, research team leads, and nominal lead of the relevant user group (e.g: SAMU ambulance staff, SAMU dispatch staff, Facility staff, and MoH/RBC). This team shall agree further development that are needed, if any.

If further development is needed, the interfaces shall undergo another cycle of user testing, as described above until it is agreed that the user interfaces are optimal; estimated 3-5 cycles are needed.

### *2. Summative evaluation*

Before deployment of the interactive systems, a summative evaluation will be conducted. Participant groups and numbers will be as above.

Longer sequences of tasks, based on the scenarios, will be constructed to evaluate the pace of work in realistic settings. As with the previous evaluations, participants will be asked to perform a think aloud after completing each task and the NASA-TLX for groups of tasks.

Data capture and analysis will proceed as detailed above.

After the information from the evaluation is integrated, a meeting will be held to discuss the results with all the relevant stakeholders – including representatives from SAMU, RBC, MoH, facility leaders, RWBuild, and the academic team.

### 3. Testing of pre-final interfaces by all users

Once the user interfaces are agreed to be optimal, potential users will be trained on the system and requested to complete a survey on the appropriateness, acceptability and feasibility and usability of the user interfaces using validated tools.

Along with survey responses, data will be collected on participant demographics (age and sex) and type of work (SAMU ambulance staff, dispatch staff, facility staff, or MoH/RBC). No personal information will be collected

Data analysis

Each survey tool has a scoring system which will be used to summarise results across all participants. Results will be described, as appropriate, as measure of central tendency and spread. Data will be disaggregated by interface.

Use of results

A brief report of the results of acceptability, appropriateness, feasibility, and usability of the interfaces will be combined with a report of the user test and training evaluation scores after completion of training (see Objective 2). This will be shared with the research team, software developers, and SAMU/RBC who will meet to discuss necessity for:

1. Further modification of the user interfaces (which will necessitate further development and testing (see above objective 1 stage 2 and objectives 1 and 2 integration)
2. Further modification of the training tools (see objective 2, below)

# **Objective 1, stage 3. Algorithm development**

An expert committee will be formed to oversee development of the DDSA and agree which destination facility is optimal for a range of different scenarios.

The algorithms which form the DDSA will be developed by the data science team using iterative cycles of development and testing. We will create a simulator to fine-tune this using iterative cycles based upon existing/baseline data (on facility readiness, patient location, and triage status) to ensure it is reliable to roll out. We will run further simulations post-deployment to further ensure that it sends patients to hospitals matching their needs. This may result in fine-tuning the algorithm over the first few weeks of deployment.

The estimated number of cases to train the algorithm is ~1000 (combined with expert validation to ensure that the training cases are making the correct decision).

## KPIs

The KPIs are that the patient is sent to the hospital that has the necessary facilities with the shortest journey time. Crucially, we will ascertain the proportion of patients that the current system correctly matches to facilities, allowing us to ensure that in addition to being more efficient, our algorithm is at least as good at allocation locations as humans.

We will not account for other factors in the fitness-function (for example, time taken to treat a patient) firstly, as data on these are not currently available, and secondly, because these are not considered in the current human-based decision-making process. In time, and for future research applications, they may be possible to consider.

Key tests include correct matching of patient to facility by the DDSA (with the DDSA’s decision being compared with that of consensus from a panel of stakeholders including SAMU ambulance staff and dispatchers, as well as facility staff).

Data used to develop scenarios to test the DDSA against stakeholders will be extracted from the existent trauma registries and SAMU records.

Criteria for successful development and readiness to deploy will be finalised in stakeholder workshops, however, investigators have indicated preliminary criteria of ≥90% for software “accuracy”.

**Analysis** will include:

1. comparisons of the performance of each iteration of the DDSA software against stakeholder agreed decisions.
2. Formal analysis will show the % agreement with Cohens Kappa used for inter-rater variability

## Iterative development

As the project progresses, more data will become available on the DDSA decisions, whether these are overridden or not by dispatch and what alternative facility dispatch selected, and why. This information will be used to fine tune the algorithm.

## System Risk testing

Prior to roll-out, basic tests of the full system will be done to describe:

- % of hospital decisions received by ambulance crews within 2 minutes of being sent by the DDSA.
- % of all information sent and received by the DDSA within 2 minutes

Other system risk tests will include:

*Compliance performance* – is the software running as expected, as per software developers’ criteria

*Scale and conflicts* - can the system handle multiple calls at once – the software will be tested with 1,3,5, and 10 patient’s data being inputted at once by multiple users.

*Priority* – assessing which requests go first if multiple data are being inputted at the same time; what does the system prioritize, and does it get this right?

*Resource constraints* – what are the impacts of equipment and tech resource constraints? Does the internet bandwidth match what the system needs and for what % of time.

Once the complete system is developed and has undergone and passed development testing, this will be shown to SAMU leadership/RBC/MoH for their approval. It will be then tested by users in simulations prior to roll out after competencies are exceeded and final approval is given by SAMU leadership/RBC/MoH.

# **Appendix 4: Objective 2 – Develop training materials and conduct staff training and testing in a classroom setting.**

Objective 2 will proceed in 2 stages: 1. rapid development of training materials and 2. delivery of training and user testing). Analysis of the process will be done to maximise the potential to transfer materials to support roll-out in other national or international settings.

# **Objective 2, stage 1: Development of training materials**

## Summary

Materials will be developed after user interfaces have reached the pre-final stage by the research team, SAMU leaders, and staff at The Rwanda Biomedical Centre and be specific to each user interface:

1. SAMU Ambulance crew
2. SAMU Dispatch
3. Facility staff

For SAMU Ambulance crews training will be given on

1. using the user interface
2. refresher triage training (based upon current local materials and those from the CDC guidelines for field triage [10], [11].

Training material format

Materials will consist of

1. a training booklet (including Standard Operating Procedures for each user interface)
2. lecture materials.
3. quick guidelines

Testing materials

We will develop a computer simulation program for training and testing using mock scenarios, we will additionally run OSCE stations for SAMU ambulance crews for inputting of triage data and for facility staff inputting readiness data.

## Methods for development and pilot testing of training materials

All materials developed will be pilot-tested with a small group of < 5 users (dispatch, ambulance staff, and facility staff) to ensure acceptability, appropriateness, and relevance; these outcomes will be assessed in FGDs with each user-group.

Results of these FGDs will be produced using rapid qualitative methods (see below) by the research team and used to adjust materials [12], [13].

The process will be iterative until the research team and users agree that materials are ready for use.

Data capture

Age, sex and employment role will be captured from participants of the pilot training.

No personal identifying information will be collected.

FGDs will be recorded for transcription, translation (if necessary) and analysis. Participants will be asked to not identify themselves during the recorded discussion.

Data analysis

We will describe:

- the training and test materials developed
- the process of development
- the number of iterations required to develop the material
- the results of the rapid analysis of the FGDs specifically looking for themes around acceptability, appropriateness, and relevance of the training materials and suggestions for change [12], [13].

# **Objective 2, stage 2: Delivery of training and testing user competencies**

## Training delivery

Training of users of Rwanda912 will be done using materials developed above, and cover all use-cases. It will consist of reading materials, lectures, practical skill sessions, and computer simulations. The computer simulations will show interfaces only that the particular user needs to interact with – these will be programmed such that all prior necessary information to allow each use case is pre-loaded.

Refresher training will also be given on patient assessment and triage at the scene based on pre-existing SAMU tools and guidance.

Training will be delivered by staff at The Rwanda Biomedical Centre, supported by the research team.

Training will be developed and delivered in Kigali and delivered in Musanze at a later stage – see Gantt chart.

There are 3 training packages:

1. Staff in facilities will receive up to 1-week training on inputting facility readiness data.

2. Ambulance staff will be given 1-2 weeks training on patient triage (which, as noted, will be based upon current protocols) inputting triage data into the platform, and extracting destination facility location.

3. Dispatch staff will be given up to two-weeks training on inputting patient location data, use of the override, extraction of reports, and use of a backup manual logic tree in case of DDSA failure.

Any staff who fails competency testing will undergo repeat training, tailored to their needs.

### Assessments of delivery of and utility of training

All trainees will be asked to complete a survey based upon Kirkpatrick’s level 1 training score [16]

## Testing user competency

### Development of material to be used in user competency testing

*SAMU Ambulance Crews*

Ability to triage patients and input triage data into mock interfaces will be tested in a classroom setting by use of an Objective Structured Clinical Examination (OSCE)-type process with actors as patients demonstrating various common trauma clinical scenarios. Scenarios will be developed which present a spectrum of injuries, based upon those which we have previously used to test clinical competencies in Malawi, and real patient data extracted from the trauma registry.

Up to 8 OSCE station scenarios will be developed by the research and SAMU team.

Ability to extract patient and facility location will be tested for each scenario.

*Dispatch*

Mock interfaces will be developed to test ability of dispatch to input information on patient location, view DDSA information on facility location, use the override, and justify that use. Mock scenarios for dispatch will be based upon historical data collected by SAMU.

Ability to extract reports of data from Rwanda912 will also be tested using simulated reports.

Correct allocation of facility using manual backup logic trees will be based upon simulated scenarios and allocation compared with that of consensus from a panel of stakeholders.

*Facility staff*

Testing materials will consist of daily reports of facility readiness based on information from actual daily reports. Mock interfaces will be developed for staff to input information and extract reports, if requested in workshops (objective 1)

###### Delivery of user competency testing

Testing will centre on competence of use of the relevant interface to input and extract data (all users – completeness and accuracy of data inputted to the interface relevant to their role) or conduct of triage (ambulance staff only).

It will be done at the end of each training period.

*SAMU Ambulance Crews*

Development of materials is described above.

An observer at each OSCE station will observe any issues that crew members have with using the software (recording observation notes). They will present a scenario of the patient and respond to any questions that the ambulance crew have regarding patient vitals for triage but will not assist the crew member in using the software or offering hints about how to conduct triage. They will record the facility that the ambulance crew has stated that they should take the patient to.

Competency will be scored at each OSCE station as the number of fields completed with accurate information as a proportion of the number of fields which should have been completed for each scenario (note that some scenarios may not require all fields to be completed). Included in the score is the recording of the facility.

*SAMU Dispatch*

Ability of users to extract reports will be tested by requesting users record information from those reports into a test sheet.

*Facility staff*

Facility staff will be tested on their ability to input accurate information and extract reports based upon real daily facility readiness reports. Data will be shown in a simulated interface and tested using an OSCE format.

Target competencies

Target scores will be agreed in the consensus workshops conducted in Objective 1. Preliminary discussions have indicated that users may be expected to score ≥90% on completeness and accuracy of data inputted to their relevant interface; those scoring less will undergo further training until competencies are achieved.

Analysis of user competency

We will describe average (and SD) competency scores amongst each user group, the number of users who fail to achieve competency.

Data will be disaggregated by type of user.

Other summary results

We will describe the number of iterations of training required to achieve competency in those who fail.

Use of results

Results from testing will be integrated into a report with that from testing of the user interfaces (described above – testing of use of pre-final interface with all users)

# **Appendix 5: Objective 3 - Conduct mock field-trials and roll out of the intervention**

# **Objective 3 stage 1: Sand-box scenario testing**

Usage of the complete active platform will be tested by all users in classroom/computer-based simulated scenarios based on data from real patients from the trauma registry and SAMU historical data with the system operational in a test-server/ “sandbox”.

Scenarios will be developed by the research team in partnership with SAMU/RBC/MoH.

Approximately 200 scenarios will be tested over the course of 1 month to ensure that all users have chance to experience the complete system.

To test each scenario will require simultaneous testing with members from facilities, dispatch, and ambulance crews. This should occur during protected (non-service provision) time

Data capture

1. *User competency* (the completeness and accuracy of information inputted) will be assessed in all users (comparing data entered by users extracted from the software testing server with that scripted into the scenarios)
2. *NASA-TCX* tool will be used to capture data on usability
3. *FGDs* with all users (separate groups of ~5 users for SAMU dispatch, SAMU ambulance crews, facility staff) on experiences with the system, or barriers or facilitators to its use (see qualitative methods, below).

Age, sex and employment role will be captured from participants. No personal identifying information will be collected.

FGDs will be recorded for transcription, translation (if necessary) and analysis. Participants will be asked to not identify themselves during the recorded discussion.

1. *Software reliability (maintenance)/functioning* will be assessed by real time capture of data by observers of users with observers looking for user-reported issues on software malfunction. Any such issues will be recorded. Observers will explore with the user to determine if the issue is a software or user issue or both.

Data analysis

1. *User competency* will be described as number [%] of form-fields filled and number [%] of form-fields filled in accurately, averaged (and standard deviation) across all users. Results will be shown overall and by user role (SAMU staff, Ambulance, Dispatch, Facility)/interface
2. *NASA-TDX* will be scored as recommended in guidelines and shown overall and by user role (SAMU staff, Ambulance, Dispatch, Facility)/interface.
3. *FGDs* will be analysed using rapid qualitative methods by the research team and presented by user type.
4. *Software reliability*/*functioning*

- results will be captured as the numbers of users experiencing issues related to software functioning and as a description of the type and number of these issues, whether these resulted in adaptions to the software, and the number of adaptions performed.
- *DDSA reliability* - Correct facility decision made by the DDSA will be done as per Objective 1 stage 3 – i.e: compared to a panel of stakeholder experts, with descriptions of % agreement and Cohen’s kappa.

Data use

1. At the end of each day of testing a report will be produced of software reliability/functioning issues recorded. These will be discussed with the research and development teams for decision making on actions to be taken regarding development fixes or adjustments to training.
2. A weekly report on competency and system usability scale scores will be compiled and fed back to the research team/SAMU/RBC/MoH for decision making on adjustments to training and when to progress to the Mock Trauma Scenario phase.
3. Once a predetermined (at the workshops by SAMU/RBC/MoH leadership) level of user competency and software reliability/functioning has been reached, the system will be tested during mock trauma scenarios.

# **Objective 3 stage 2 - Mock Trauma Scenarios**

Mock trauma scenarios will be based on real patients from the trauma registry (and/or Primary Trauma Care Guidelines [17] and use dummy patients (actors) at different locations in Kigali. All system users participating in the scenarios will enter and extract information in as close an approximation to real world as possible, apart from ambulance crews will not remove the “patient” from the scene and “ambulances” may be replaced with taxis.

Observers will be stationed at each “patient”, in each “ambulance”, in dispatch, and in facilities to record observations on user interaction with software and times that data are entered/received/extracted.

Scenarios will run over the course of 1-2 weeks and aim to involve all individual users of the system.

Aims are two-fold

1. To test user competency
2. To test software reliability/functioning in “real world”

Data capture

1. *User competency* (the completeness and accuracy of information inputted) will be assessed in all users (comparing data entered by users extracted from the software testing server with that scripted into the scenarios).
2. *NASA-TCX* tool will be used to capture data on usability
3. *Software reliability/functioning* will be assessed by real time capture of data by observers of users with observers looking for user-reported issues on software “malfunction”. Any such issues will be recorded. Observers will explore with the user to determine if the issue is a software or user issue.
4. *FGDs* with all users (separate groups of ~5 users for SAMU dispatch, SAMU ambulance crews, facility staff, and RBC staff) on experiences of the mock trauma scenarios (see qualitative methods, below).

Age, sex and employment role will be captured from participants. No personal identifying information will be collected.

FGDs will be recorded for transcription, translation (if necessary) and analysis. Participants will be asked to not identify themselves during the recorded discussion.

Data analysis

1. User competency will be described as number [%] of form-fields filled and number [%] of form-fields filled in accurately (out of all form fields which should have been completed for a particular scenario). Results will be averaged across users and shown overall and by user role (SAMU staff, Ambulance, Dispatch, Facility)/interface
2. *NASA-TDX* will be scored as recommended in guidelines and shown overall and by user role (SAMU staff, Ambulance, Dispatch, Facility)/interface.
3. *FGDs* will be analysed using rapid qualitative methods by the research team and presented by user type [12], [13].
4. Software reliability/functioning results will be captured as the numbers of users experiencing issues and a description of the type and number of these issues, whether these resulted in adaptions to the software, and the number of adaptions performed
5. DDSA reliability - Correct facility decision made by the DDSA (done as per Objective 1 stage 3 – i.e: compared to a panel of stakeholder experts, with descriptions of % agreement and Cohen’s kappa).

Data use

1. At the end of each day of testing a report will be produced of software reliability issues recorded. These will be discussed with the research and development teams for decision making on actions to be taken regarding development fixes or adjustments to training (for user issues reported).
2. A report on competency and system usability scale scores will be compiled and fed back to the research team/SAMU/RBC/MoH for decision making on adjustments to training.
3. A complete report of the mock field trials including user competency, NASA-NTX, and software reliability will be submitted to MoH/RBC/SAMU for decision making on roll out or further development and training.

- If roll out is decided, the software will be rolled out in Kigali (and Musanze at a later stage).
- If further development and training is required, these will be delivered and further sand-box scenario testing done until a threshold of competency/user score/software reliability is passed to allow a limited repeat of mock field trials.

## Roll out

Roll out will be done over the course of 1 month, with the current system being replaced (in each study site) with the new system and all study investigators being on-hand to discuss and resolve any problems. All issues and solutions will be recorded and reported.

# **Appendix 6: General Qualitative methodologies**

## Summary

Focus Groups will contain 5-7 members of each user type.

Age, sex and employment role will be captured from participants. No personal identifying information will be collected.

FGDs will be recorded for transcription, translation (if necessary) and analysis. Participants will be asked to not identify themselves during the recorded discussion.

Each FGD will last around 1h.

Qualitative data will be collected throughout the study.

We will use rapid qualitative analysis techniques in Objectives 1 & 2 to inform iterations of development of the Rwanda912 system or training materials, as previously described. In other objectives, qualitative methods will be used to develop in-depth explorations of perceptions of and experiences with Rwanda912. In general, we will use FGDs with each separate user group (including patients, after intervention roll-out). In-depth interviews (IDIs) will be conducted to supplement FGD findings with respondents who have expressed particularly compelling views in the FGDs. They will also be used to capture data from policy makers who may not be able to gather for FGDs. We will use rapid qualitative analysis methods when results from qualitative analyses are needed quickly to inform development
