# Supplementary material for: Developing and integrating a destination decision support algorithm into an innovative electronic communication platform to improve injury care service coordination in Rwanda: the Rwanda912 study protocol
Source: BMJ Open. 2025 Jun 27;15(6):e102355. doi: 10.1136/bmjopen-2025-102355 (PMC12207105; doi:10.1136/bmjopen-2025-102355)
Supplement: Supplementary file 4 [file bmjopen-2025-102355.reviewer_comments.pdf]

PEER REVIEW HISTORY

BMJ Open publishes all reviews undertaken for accepted manuscripts. Reviewers are asked to complete a checklist review form and are provided with free text boxes to elaborate on their assessment. These free text comments are reproduced below.

ARTICLE DETAILS

Title (Provisional)

Developing and integrating a destination decision support algorithm into an innovative electronic communication platform to improve injury care service coordination in Rwanda: The Rwanda912 study protocol

Authors

Nishimwe, Aurore; Rwanda912, RIGHT GROUP

This article was not externally reviewed at BMJ Open. Protocols that have been independently assessed prior to submission to BMJ Open are usually fast-tracked to publication on the grounds that further substantial changes will not be possible. This independent assessment will usually be external, independent review for both a substantial grant award from a non-commercial or government funder and ethics approval.
